# Supplementary material for: Splice-Junction-Based Mapping of Alternative Isoforms in the Human Proteome
Source: Cell Rep. Author manuscript; Available in PMC 2020 Jan 15. (PMC6961840; doi:10.1016/j.celrep.2019.11.026)
Supplement: 2 [file NIHMS1546469-supplement-2.zip › DF1/PXD006675/RightAtrium/RightAtrium_46_MINK1_AATAAGAGAAEAAGEGAAAAAGGHAGSAAGGGAAAG.pdf]

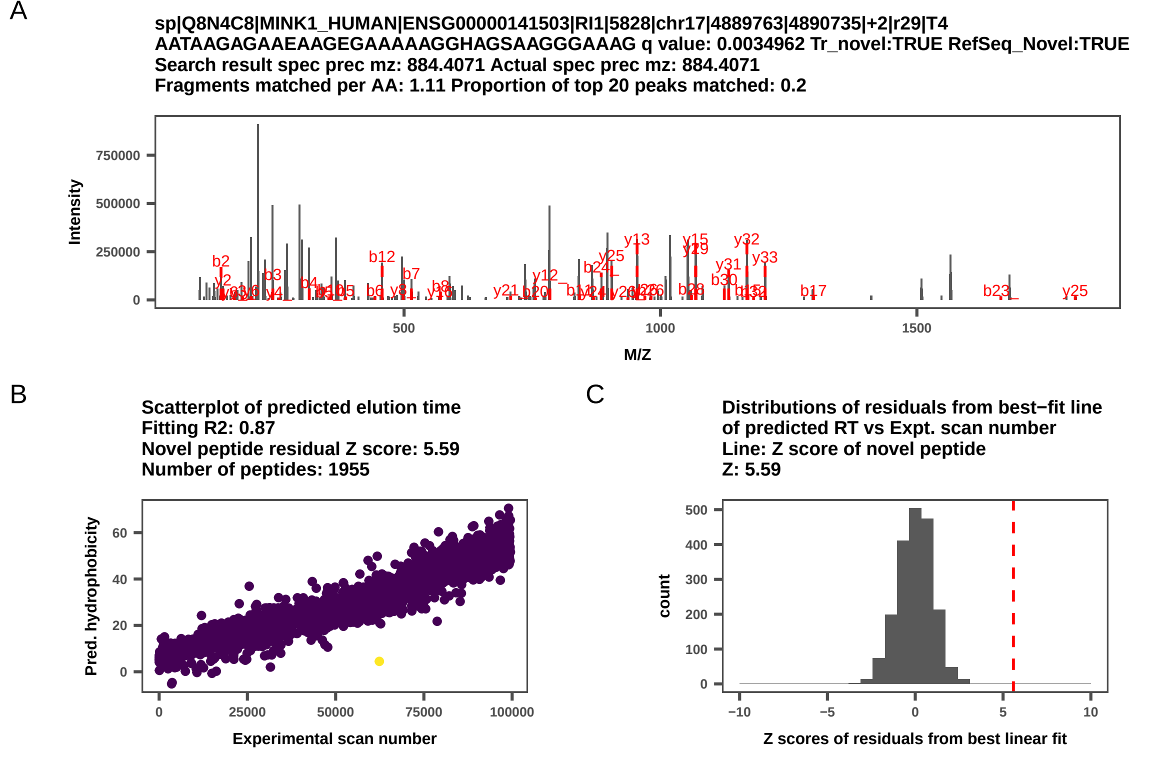
A
sp|Q8N4C8|MINK1_HUMAN|ENSG00000141503|RI1|5828|chr17|4889763|4890735|+2|r29|T4
AATAAGAGAAEAAGEGAAAAAGGHAGSAAGGGAAAG q value: 0.0034962 Tr_novel:TRUE RefSeq_Novel:TRUE
Search result spec prec mz: 884.4071 Actual spec prec mz: 884.4071
Fragments matched per AA: 1.11 Proportion of top 20 peaks matched: 0.2
Intensity
750000
500000
250000
0
500
1000
1500
M/Z
b2
y2
b3
y4
b4
b12
b6
y8
b7
b8
y21
b20
y12
b24
y25
y13
y15
y29
y32
y31
y33
b30
b28
b17
b23
y25
B
Scatterplot of predicted elution time
Fitting R2: 0.87
Novel peptide residual Z score: 5.59
Number of peptides: 1955
Pred. hydrophobicity
60
40
20
0
0
25000
50000
75000
100000
Experimental scan number
C
Distributions of residuals from best−fit line
of predicted RT vs Expt. scan number
Line: Z score of novel peptide
Z: 5.59
count
500
400
300
200
100
0
−10
−5
0
5
10
Z scores of residuals from best linear fit
